# Supplementary material for: Distinguishing between parallel and serial processing in visual attention from neurobiological data
Source: R Soc Open Sci. 2020 Jan 29;7(1):191553. doi: 10.1098/rsos.191553 (PMC7029944; doi:10.1098/rsos.191553)
Supplement: Information on data and results from the simulation study [file rsos191553supp1.pdf]

Supporting information

This is Supporting Information for the paper **Distinguishing between parallel and serial processing in visual attention from neurobiological data** by Kang Li, Mikiko Kadohisa, Makoto Kusunoki, John Duncan, Claus Bundesen, and Susanne Ditlevsen.

Computer code and data is available as separate files in supplementary material.

I: Data

To get an overall idea of the sample sizes in the experimental data, histograms in Fig S1 show the average number of trials per condition over the 48 sessions, the average number of simultaneously recorded neurons per trial over sessions, and a histogram over neurons per trial. Single trials have between 1 and 11 neurons recorded. Trials with only one recorded neuron only contribute to obtain better estimates of base firing rates.

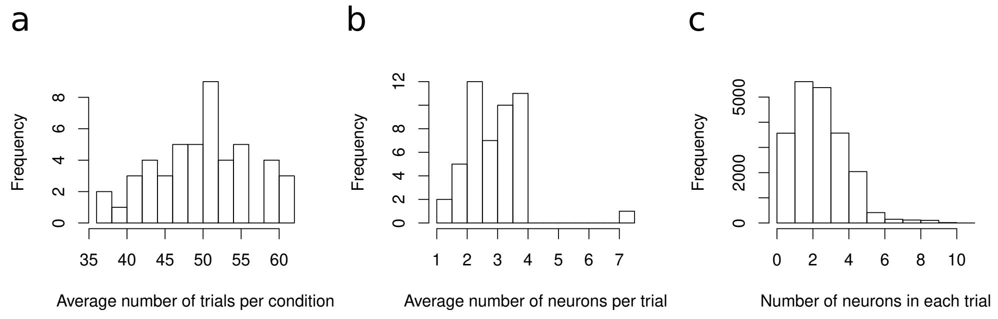

Figure S1. Data structure and sample sizes. a) Average number of trials per condition in 48 sessions. b) Average number of neurons per trial in 48 sessions. In all sessions, at least 5 neurons are recorded, however, not all enter in each trial. Histogram are based on 48 numbers (one for each session). c) Number of neurons per trial.

Fig S2 shows an example neuron, complementary to the neuron in Fig 2 in the main paper, which shows a tendency to the ipsilateral stimulus in the early stage, and later the attention is redirected to the target stimulus. Furthermore, for this neuron there is more variability between trials under the same condition.

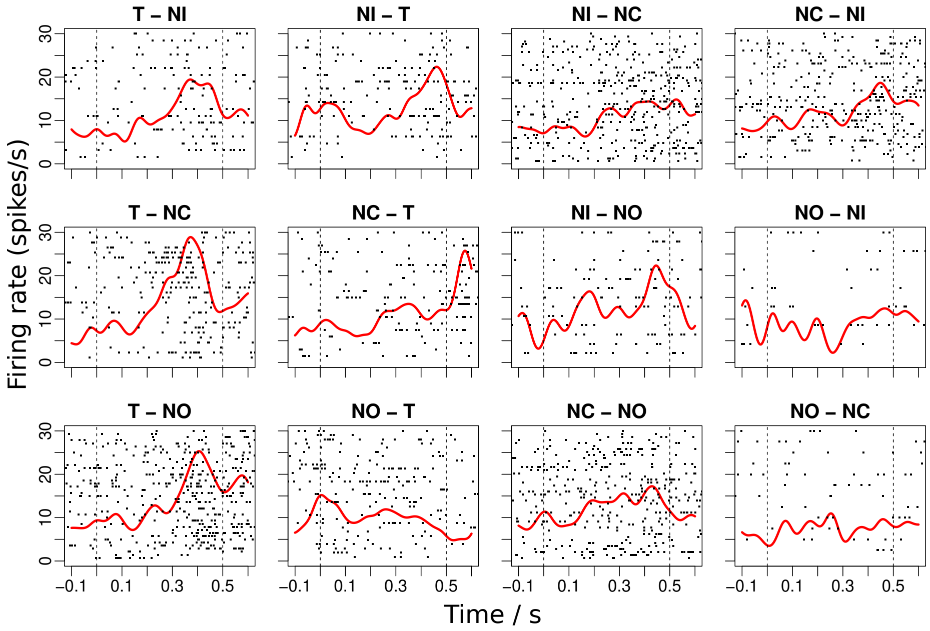

Figure S2. Raster plots of measured spike trains recorded from an example cell (mj081029a_8_0). The 12 conditions are indicated in the title of the subplot. Kernel smoothing estimates of the firing rates are shown in red. The stimulus in the left of the title indicates the stimulus of the contralateral side, and the right indicates the stimulus on the ipsilateral side with respect to the recorded neuron. The dashed lines indicate the interval of the choice phase where two stimuli are shown.

II: The simulation study

Results from the simulation study Here we report the results from the simulation study. For both the HMM and the CBM, we consider three parameter settings. In all cases, we use 10 simultaneously recorded neurons, repeated for 20 trials. The parameters, including base rates and response weights, are the same for the three cases. We consider only one stimulus condition, such that each neuron only has two base rate parameters, one for the contralateral and one for the ipsilateral sides.

The parameter values used in the simulations are shown in Table S1. For the HMM, we use a time step of $0.1s$ and a total of 10 time steps. For the CBM, we use a time step of $0.1s$ and a total of 5 time steps. Fig 5 in the main paper shows the probabilities, correlations, D^* and D_n values as functions of time. Simulated example spike trains are also shown.

We apply the model fitting to the simulated data, and the MLEs are shown in Figs S3 and S4 together with the true parameters for the HMM and the CBM. The simulation and model fitting procedure are repeated 100 times. The D^* and D_n values are computed from the parameter estimates, and are shown in Fig 6 in the main paper together with the true D^* and D_n values. Finally, we also perform decoding analysis using the estimated parameters for each trial. The D_n values from the decoding are plotted in Fig S5 together with the D_n values computed directly from the parameter estimates.

rsos.royalsocietypublishing.org R. Soc. open sci. 000000

Table S1. Parameters used to simulate data from the HMM and the CBM. Different ρ and p parameter values are used at different time steps t. Firing rates and weight values for the 10 simulated neurons are the same in the two models. The base firing rate is denoted by $r_{i,j}$ for stimulus i and neuron j. The contralateral stimulus is represented by $i = 1$ and the ipsilateral stimulus by $i = 2$. The memory weights are the same for all neurons, denoted by β.

	Parameters	
	**Hidden Markov Model**, $\alpha = (0.91, 0.45, 0.15)$	
	λ	Γ
Case 1	$[0.3\ \ 0.65\ \ 0.05]^T$	$\begin{bmatrix} 0.95 & 0.02 & 0.03 \\ 0.1 & 0.8 & 0.1 \\ 0.5 & 0.2 & 0.3 \end{bmatrix}$
Case 2	$[0.5\ \ 0.4\ \ 0.1]^T$	$\begin{bmatrix} 0.3 & 0.6 & 0.1 \\ 0.1 & 0.3 & 0.6 \\ 0.01 & 0.01 & 0.98 \end{bmatrix}$
Case 3	$[0.7\ \ 0.15\ \ 0.15]^T$	$\begin{bmatrix} 0.2 & 0.1 & 0.7 \\ 0.05 & 0.9 & 0.05 \\ 0.7 & 0.1 & 0.2 \end{bmatrix}$
	**Correlated Binomial Model**	
	$\rho_t\ (t = 1, \ldots, 5)$	$p_t\ (t = 1, \ldots, 5)$
Case 1	$(0.6, 0.6, 0.3, 0.15, 0.1)$	$(0.7, 0.65, 0.72, 0.75, 0.75)$
Case 2	$(0.2, 0.2, 0.3, 0.4, 0.4)$	$(0.65, 0.7, 0.75, 0.8, 0.8)$
Case 3	$(0.3, 0.3, 0.4, 0.45, 0.5)$	$(0.6, 0.55, 0.4, 0.3, 0.3)$
	**Base rates**	
	$r_{i,j}$ ($i = 1, 2$ and $j = 1, \ldots, 10$)	
contralateral	$(80, 80, 80, 90, 60, 70, 80, 70, 50, 40)$	
ipsilateral	$(20, 10, 30, 10, 30, 20, 10, 20, 10, 20)$	
	**Memory weights**	
	β	
	$(-2, -0.5, 0, 0.5, 0.2, 0.1, 0.1, 0.1, 0.05, 0.05)$	

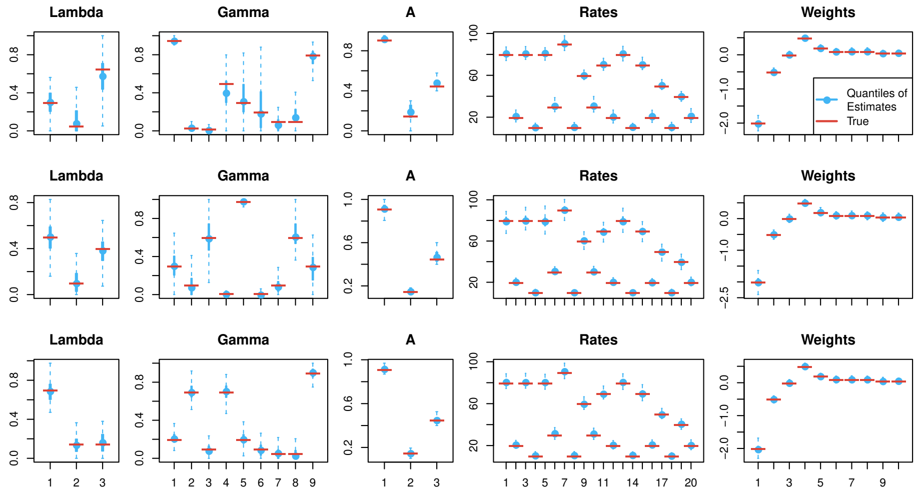

Figure S3. Simulation study: Parameter estimates of the hidden Markov model. The estimates are shown as quantiles of the 100 repetitions. The dashed lines represent the full $0-100\%$ quantiles, and the solid lines represent the $25\%-75\%$ quantiles. The central dot is the median. The red lines are the true values used in the simulation. The upper, middle and lower panels represent the three cases.

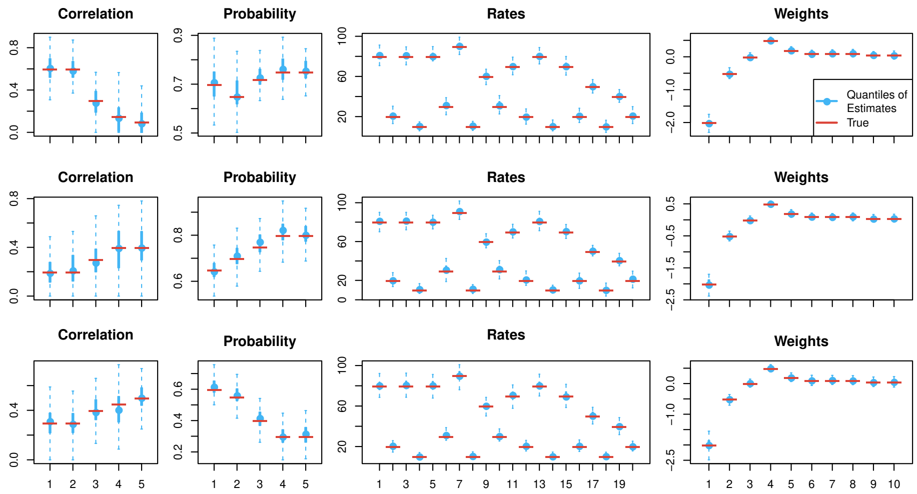

Figure S4. Simulation study: Parameter estimates of the correlated binomial model. The estimates are shown as quantiles of the 100 repetitions. The dashed lines represent the full $0-100\%$ quantiles, and the solid lines represent the $25\%-75\%$ quantiles. The central dot is the median. The red lines are the true values used in the simulation. The upper, middle and lower panels represent the three cases.

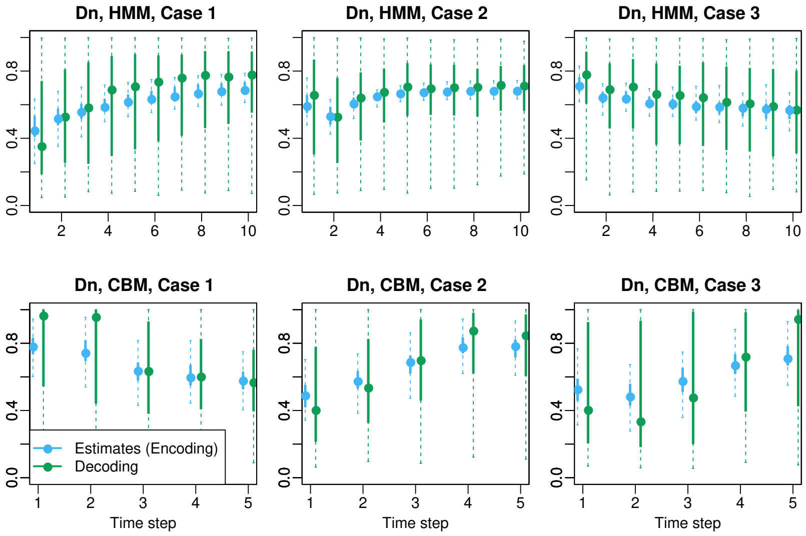

Figure S5. Simulation study: Deviation statistic values D_n obtained from decoding or encoding analysis. Values are calculated from estimates (encoding, blue) or from decoding analysis (green) and are shown as quantiles of the 100 repetitions. The dashed lines represent the full $0 - 100\%$ quantiles, and the solid lines represent the $25\% - 75\%$ quantiles. The dots are the medians.
